# Moesin and Stress-Induced Phosphoprotein-1 Are Possible Sero-Diagnostic Markers of Psoriasis

**DOI:** 10.1371/journal.pone.0101773

**Published:** 2014-07-10

**Authors:** Hideki Maejima, Ryo Nagashio, Kengo Yanagita, Yuko Hamada, Yasuyuki Amoh, Yuichi Sato, Kensei Katsuoka

**Affiliations:** 1 Department of Dermatology, Kitasato University School of Medicine, Sagamihara, Japan; 2 Department of Applied Tumor Pathology, Kitasato University Graduate School of Medical Sciences, Sagamihara, Japan; 3 Department of Molecular Diagnostics, School of Allied Health Sciences, Kitasato University, Sagamihara, Japan; University of Saarland Medical School, Germany

## Abstract

To identify diagnostic markers for psoriasis vulgaris and psoriatic arthritis, autoantibodies in sera from psoriasis vulgaris and psoriatic arthritis patients were screened by two-dimensional immunoblotting (2D-IB). Based on 2D-IB and MADLI TOF/TOF-MS analyses, eleven proteins each in psoriasis vulgaris and psoriatic arthritis were identified as autoantigens. Furthermore, serum levels of moesin, keratin 17 (K17), annexin A1 (ANXA1), and stress-induced phophoprotein-1 (STIP1), which were detected as autoantigens, were studied by dot blot analysis with psoriasis patients and healthy controls. The levels of moesin and STIP1 were significantly higher in sera from patients with psoriasis vulgaris than in the controls (moesin: *P*<0.05, STIP1: *P*<0.005). The area under the curve (AUC) for moesin and STIP1 between patients with psoraisis vulgaris and controls was 0.747 and 0.792, respectively. STIP1 and K17 levels were significantly higher in sera from patients with psoriatic arthritis than in those with psoriasis vulgaris (*P*<0.05 each). The AUC for STIP1 and K17 between patients with psoriatic arthritis and psoriasis vulgaris was 0.69 and 0.72, respectively. The STIP1 or moesin, CK17 serum level was not correlated with disease activity of psoriasis patients. These data suggest that STIP1 and moesin may be novel and differential sero-diagnostic markers for psoriasis vulgaris and psoriatic arthritis.

## Introduction

Psoriasis is a chronic, relapsing, and inflammatory skin disease that affects 2% of the Caucasian population [1]. This skin condition is histologically characterized by abnormal proliferation of keratinocytes and infiltration of immune cells, predominantly T-cells and dendritic cells, in psoriatic lesions [2]. The search for biomarkers for psoriasis has included not only those found in the skin, but also the spillover of inflammatory markers into systemic circulation. Markers of psoriasis have been used to measure disease severity, to objectively monitor treatment response, to find new targets for therapy, and to explain comorbidities in psoriasis patients [3], [4]. In this study, we detected psoriasis-associated autoantibodies by immunoblotting based on two-dimensional gel electrophoresis (2-DE) using sera from patients with psoriasis vulgaris or psoriatic arthritis as first antibodies. Identified antigens recognized with autoantibodies were further immunohistochemically assessed to confirm their stainability in psoriasis vulgaris or psoriatic arthritis tissues. Finally, serum levels of autoantigens were validated by dot blot analyses of sera from psoriasis vulgaris and psoriatic arthritis patients and healthy controls.

## Materials and Methods

### Cell lines

Normal human epidermal keratinocytes (NHK) from neonatal foreskin (Kurabo, Osaka, Japan) were grown in EpiLife medium (Cascade Biologics, Carlsbad, CA). A431 cells derived from epidermoid carcinoma (American Type Culture Collection, Manassas, VA) were grown in Dulbecco’s Modified Eagle Medium (Gibco, Grand Island, NY) supplemented with 10% fetal bovine serum.

### Ethics statement

This study was approved by the Ethics Committee of Kitasato University School of Medicine (number B10-93) and followed the Declaration of Helsinki protocols. All patients and healthy controls were approached based on approved ethical guidelines, agreed to participate in the study, and could refuse entry and discontinue participation at any time. All participants provided written consent.

### Sera

Sera from 31 patients with psoriasis vulgaris, 12 with psoriatic arthritis, and 13 healthy controls were used in this study. Sex, age, and disease duration, as well as an assessment of disease severity using the psoriasis area and severity index (PASI) and percentage of percentage of Body Surface Area involved (BSA) in patients with psoriasis vulgaris and psoriatic arthritis [5], disease activity score 28 C reactive protein (DAS28-CRP) [6], bath ankylosing spondylitis disease activity index (BASDAI) [7], swollen or tender joint counts, and involved joint counts in patients with psoriatic arthritis, were recorded. Diagnosis of psoriatic arthritis was based on the CASPAR criteria [8]. The characteristics of the 43 psoriasis patients are summarized in Table 1.

**Table 1:** Clinical characteristics of psoriasis patients.

(n)	Psoriasis vulgaris (31)	Psoriatic arthritis (12)
Mean age	55.7(28–85)	44.7(19–74)
Sex(Male:Female)	26∶5	8∶4
disease duration(year)	10.0(0.1–29)	5.7(0.8–13)
PASI	13.2(0.6–41.4)	10.1(0.7–44.1)
BSA	25.6(1–95)	13.3(1–95)
DAS28	Not detected	3.75(1.77–6.48)
BASDAI		3.57(0.3–7.6)
Swollen joint counts		4.9(0–14)
Tender joint counts		4.4(1–14)
Involved joint counts		7.9(1–23)

### Skin biopsy specimens

Thirty-three 10% formalin-fixed and paraffin-embedded skin biopsy specimens of psoriasis (25 psoriasis vulgaris and 8 psoriatic arthritis) were used in this study. Eight each of human epidermal cyst and normal human epidermis were also used as controls.

### Proteome analysis

#### Two dimensional gel electrophoresis (2-DE)

Sample preparation and the 2-DE method used in this study were described in our previous paper [9]. Proteins extracted from a mixture of NHK and A431 cell lines were separated by 2-DE. Two sets of gels were prepared for each sample, and one was transferred to a polyvinylidene fluoride (PVDF) membrane (Millipore Corp, Bedford, MA) for immunoblotting, and the other was visualized by Coomassie Brilliant Blue R-350 (CBB) staining (PhastGel Blue R, GE Healthcare, Uppsala, Sweden). Blotting membranes were blocked with 0.05% casein/TBS (0.01 mol/l Tris-HCl, pH 7.5, 150 nmol/l NaCl) for 60 min at room temperature (RT). Membranes were reacted with 100-times diluted pooled sera with three each psoriasis vulgaris or psoriatic arthritis patients with 0.0025% casein/TBS-T (TBS containing 0.1% Tween-20) as primary antibodies for 2 h at RT. Then the membranes were incubated with 1,000-times diluted horseradish peroxidase-conjugated rabbit anti-human IgG polyclonal antibody (Dako, Glostrup, Denmark) for 30 min at RT. Finally, signals were developed by Stable DAB solution (Invitrogen, Calsbad, CA).

#### Identification of autoantigens

The method of protein identification was described in our previous study [10]. In brief, protein spots that reacted with patient sera were excised from 2-DE gels and destained with 50% (v/v) acetonitrile (ACN)/50 mM NH_4_HCO_3_, dehydrated with 100% (v/v) ACN, and then dried under vacuum conditions. Tryptic digestion was performed for 24 h at 37°C in a minimum volume of digestion solution containing 10 ng/µl sequencing grade modified trypsin (Promega Corp., Madison, WI) and 25 mM NH_4_HCO_3_. After incubation, digested protein fragments eluted in solutions were collected, and gels were washed once in 50% (v/v) ACN/5% trifluoroacetic acid and collected in the same tube. Then, solutions were measured by MALDI-TOF/TOF MS (autoflex-III TOF/TOF; Bruker Daltonik GmbH, Bremen, Germany). Fragment ion spectra from MS and MS/MS were submitted to MSACOT (http://www.matrixscience.com, accessed 21 May 2014) for a database search and the corresponding proteins were indentified employing the following database: IPI human 20091026 (86379 sequences; 347407090 residues, http://www.ebi.ac.uk/services human; http://www.matrixscience.com, accessed 21 May 2014).

### Immunohistochemical staining

Four-µm-thick tissue sections made from 10% formalin-fixed and paraffin-embedded psoriasis tissues and normal human skins were used. Deparaffinized and rehydrated sections were treated with 0.3% hydrogen peroxidase for 10 min. Then, antigens were retrieved for 5 min with 10 mM citrate buffer (pH 6.0) in a microwave oven, and the sections were maintained until the solution decreased to RT. After washing with 100 mM phosphate buffered saline (pH 7.4; PBS) for 5 min and blocking non-specific protein binding with 2% normal swine serum (Dako Carpinteria, CA) for 10 min, the sections were incubated with ANXA1 polycloncal antibody (1∶200; Abcam, Cambridge, UK), anti-K17 polyclonal antibody (1∶100; Abcam), anti-STIP1 monoclonal antibody (1∶600; Abnova, Taipei City, Taiwan), or anti-moesin monoclonal antibody (1∶300; Abnova) for 60 min each at RT. Then the sections were incubated with ChemMate ENVISION reagent (Dako) for 30 min at RT. Finally, the sections were visualized with Stable DAB solution and counterstained with Mayer’s hematoxylin (Wako Pure Chemical, Osaka, Japan). The stainability of ANXA1, K17, STIP1, and moesin was scored as 0 = absent; 1 = positive cells were scattered; 2 = almost all cells were positive in epidermis.

### Dot blot analysis

#### Sample preparation

The removal of albumin and IgG from sera was performed using a ProteoExtract Albumin/IgG Removal kit (Merck, Darmstadt, Germany) according to the manufacturer’s instructions. In brief, 60 µL of each serum was diluted with 540 µL of binding buffer and allowed to pass through the column by gravity flow. The flow-through fraction was collected in a collection tube. To wash the column, binding buffer was allowed to pass through the column by gravity flow. The flow-through fraction was collected in the same collection tube. Desalting and concentration were performed by ultrafiltration. The albumin- and IgG-depleted samples were buffer-exchanged and centrifuged using 10-kDa molecular-weight cut-off ultra-filtration VIVASPIN 2 (Sartorius, Gottingen, Germany). The samples were centrifuged at 6,000×*g* at 4°C until less than 100 µL, and then the buffer was exchanged for PBS (–) with centrifugation at 6,000×*g* at 4°C until concentrated to less than 50 µL. The concentrated samples were adjusted to a final volume of 60 µL with PBS (–)

#### Micro-dot blot array

The serum levels of moesin, K17, STIP1, and ANXA1 were detected by a micro-dot blot arrayer with a 256 solid-pin configuration (Kakengeneqs Co., Ltd., Chiba, Japan). In brief, 1 µl of 20-times diluted, pre-treated serum with 0.01% Triton-X ×100 solution were spotted onto PVDF membranes. After being washed in TBS, the membranes were blocked with 2% Tween-20/TBS for 1 h at RT. Then the membranes were reacted with 4,000-times diluted first antibodies (anti-moesin, anti-K17, anti-STIP1, and anti-ANXA1) with 0.1% Tween-20/TBS for 30 min at RT. After TBS-T washing 3 times for 5 min each, the membranes were incubated with 1,000-times diluted horseradish peroxidase (HRP)-conjugated rabbit anti-mouse IgG polyclonal antibody for moesin and STIP1, or 5,000-times diluted alkaline phosphatase (AP)-conjugated goat anti-rabbit IgG polyclonal antibody for K17 and ANXA1 for 30 min each at RT. Finally, signals were developed with Immobilon Western reagent (Millipore Corp.) for HRP or PhosphaGLO AP substrate (KPL, Gaithersbrug, MD) for AP. The data were analyzed using Dot Blot Chip-System software ver.4.0 (Dynacom Co., Ltd., Chiba, Japan). The signal of each serum was presented as normalized by the positive intensity minus background intensity around the spot.

### Statistical analysis

Statistical analysis was performed using the Mann-Whitney U test. The area under the curve and best cut-off point were calculated employing ROC analysis. Results were considered significant when *P*<0.05. All P values used are two sided. Analysis was performed using StatFlex version 6.0 software (Artech Co., Ltd., Osaka, Japan).

## Results

### Identification of autoantigens

**Figure 1:**
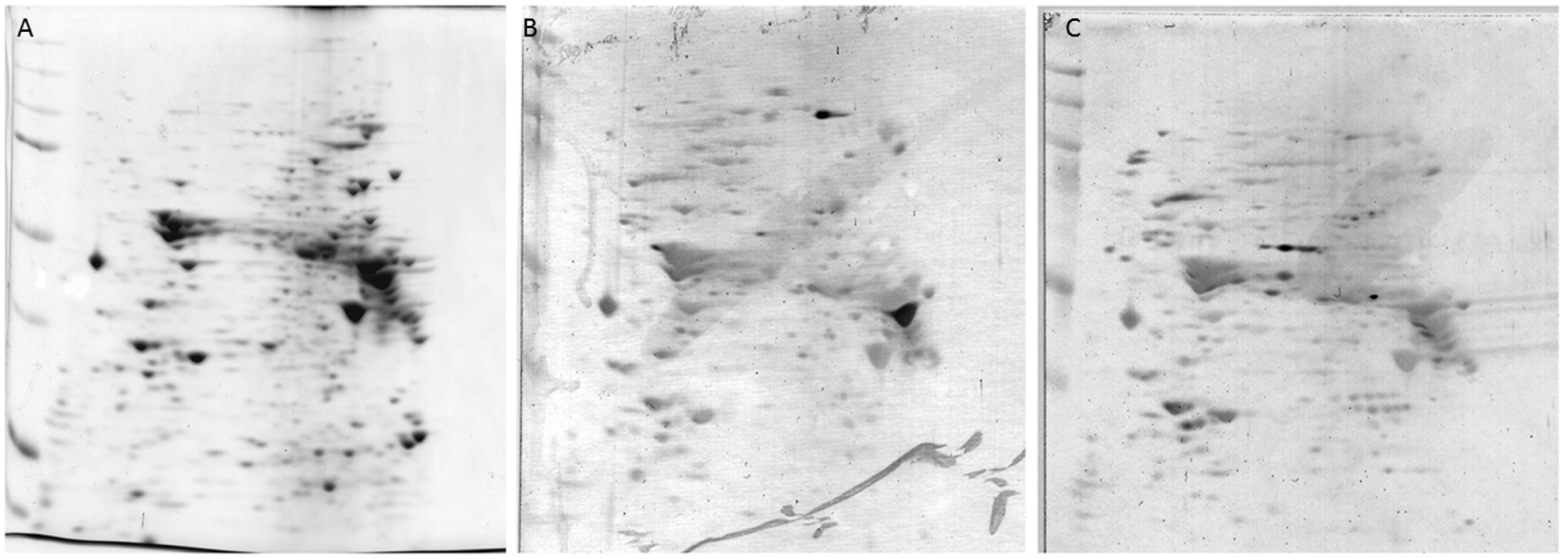
Detection of Autoantibodies by 2D-immunoblotting. (**A**) The 2-DE protein pattern after CBB staining. Immunoblot analysis with mixed sera from patients with psoriasis vulgaris (**B**) and psoriatic arthritis (**C**) as primary antibodies, respectively. Several proteins were positively detected as autoantigens in each disease.

The immunoreactivities of autoantibodies in sera from psoriasis patients were assessed by two dimensional immunoblotting (2D-IB). Representative positive protein spots on the membrane are shown in Figure 1. Thirty-seven and 33 positive spots were detected in sera from patients with psoriasis vulgaris and psoriatic arthritis, respectively. Proteins that were expressed 1.5 times higher than those in healthy controls were assumed as positive. In total, 11 proteins each for patients with psoriasis vulgaris and psoriatic arthritis were identified as autoantigens. Identified proteins are summarized in Tables 2 and 3. Two proteins each, moesin and K17 for psoriasis vulgaris, and STIP1 and ANXA1 for psoriatic arthritis, were selected for further experiments, because the expression levels of these proteins were more than twice those in healthy controls. In addition, their antibodies are commercially available.

**Table 2:** Autoantigens identified using sera from psoriasis vulgaris patients.

Gene symbol	Protein name	Molecularweight	Function	Localized	Expression raito compared with contorls
VCP	Transitional endoplasmic reticulum ATP ase	89266	ATP ase activity	Cytoplasma NucleusNucleous perinuclearregion	5.4
MSN	Moesin	67778	Cytoskeletal protein	Cytoplasma	3.9
CCT3	T-complex protein 1 submit gamma	60495	Chaperone activity	Cytoplasma	3.5
K17	Cytokerain 17	48076	Structural constituent of cytoskeleton	Cytoplasma	3.3
CS	Citrate synthase mitochondrial	51680	Actytansferase activity	Mitochondrial matrix	2.5
ACTR3	Actin-related protein 3	47341	Structural constituent of cytoskeleton	Cytoplasma	2
AHCY	Adenosylhomocysteinase	47685	Hydrolase activity	Cytoplasma	1.9
ANXA1	Annexin A1	54355	Calucium ion binding	Cytoplasma	1.9
EEF1A1	Elongation factor 1-alpha 1	50109	Transcription regulatoractivity	Cytoplasma	1.8
EIF4A2	Eukaryotic initiation factor 4A-II	46373	Transcription regulatoractivity	Cytoplasma	1.7
TKT	Transketolase	67385	Tranferase actvity,transferring aldehydeor ketonic proup	Cytoplasma	1.5

**Table 3:** Autoantigens identified using sera from psoriatic arthritis patients**.**

Gene symbol	Protein name	Molecular weight	Function	Localized	Expression raito compared with contorls
STIP1	Stress-induced- phosphoprotein 1	62599	Reseptor signalingcomplex scaffoldactivity	Nucleus	7.7
ACTN4	Alpha-actinin-4	104788	Structual constituent of cytoskelenton	Cytoplasma	4.2
ANXA1	Annexin A1	38690	calucium ion binding	Plasma membrane	2.3
SFN	14-3-3 protein sigma	27757	Reseptor signalingcomplex scaffoldactivity	Cytoplasma	2.2
LMNA	Lamin-A/C	74095	Structual molecule activity	Nucleus	2.1
NEURL4	Neuralized-like protein 4	166802	Unknown	Unknown	2
ATP5A1	ATP synthase submit alpha, mitochondrial	59714	Transporter activity	Mitochondrion	1.7
GAPDH	Glyceraldehyde-3-phophate dehydrogenasis	36030	Enzyme; Dehydrogenase	Cytoplasma	1.6
CYCS	Cytochrome C	11741	Catalytic activity	Mitochondrion	1.6
GLUD1	Glutamate dehydorgenase 1, mitochondrial	61359	Catalytic activity	Mitochondrion	1.5
PA2G4	Proliferation-associated protein 2G4	43759	Tracription regulatoractivity	Nucleus	1.5

### Immunohistochemical staining for moesin, K17, STIP1, and ANXA1 in psoriatic epidermis

**Figure 2:**
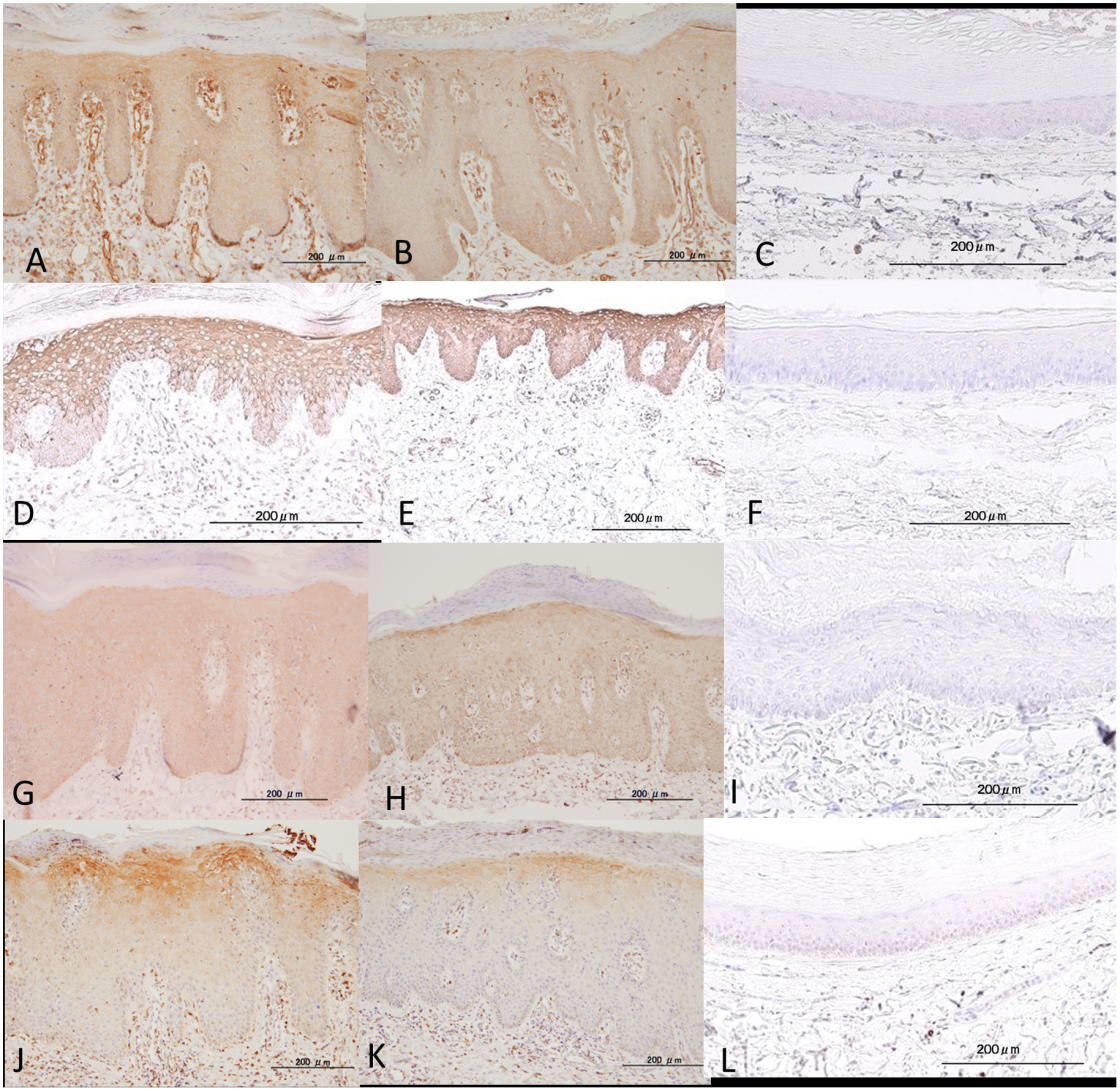
Expression of moesin (A, B, C), K17 (D, E, F), STIP1 (G, H, I), and ANXA1 (J, K, L) in psoriasis vulgaris (A, D, G, J), psoriatic arthritis (B, E, H, K), and control (C, F, I, L). Different expression levels of moesin, K17, STIP1, and ANXA1 were observed in epidermis of psoriasis vulgaris and psoriatic arthritis, but weak expressions were detected in the epidermis of normal skin. Bar = 200 µm.

Moesin expression was observed in the upper stratum spinosum and basal layer in the epidermis of 28 of 34 of psoriatic skin samples, including psoriasis vulgaris and psoriatic arthritis (Figure 2A, B), but was negative in 16 controls except one (Figure 2C). K17 expression was observed in the stratum spinosum of all epidermis samples of psoriatic skin (Figure 2D, E), and was negative in 10 of 15 controls (Figure 2F). STIP1 was diffusely positive throughout the epidermis in all psoriatic skin samples (Figure 2G, H), but was negative in 11 of 15 controls (Figure 2I). ANXA1 was positive of the upper stratum spinosum in the epidermis of all psoriatic skin samples (Figure 2J, K) but not in controls (Figure 2L). Even when positive, the expression levels were stronger in psoriatic epidermis than in controls (*P*<0.01). No significant differences between the expression levels or localization of these proteins and clinico-pathological features such as sex, age, psoriasis vulgaris vs. psoriatic arthritis, PASI score, or BSA, DAS28, BASDAI swollen or tender joint counts, and involved joint counts.

### Serum levels of moesin, K17, STIP1, and ANXA1 in psoriasis patients

**Figure 3:**
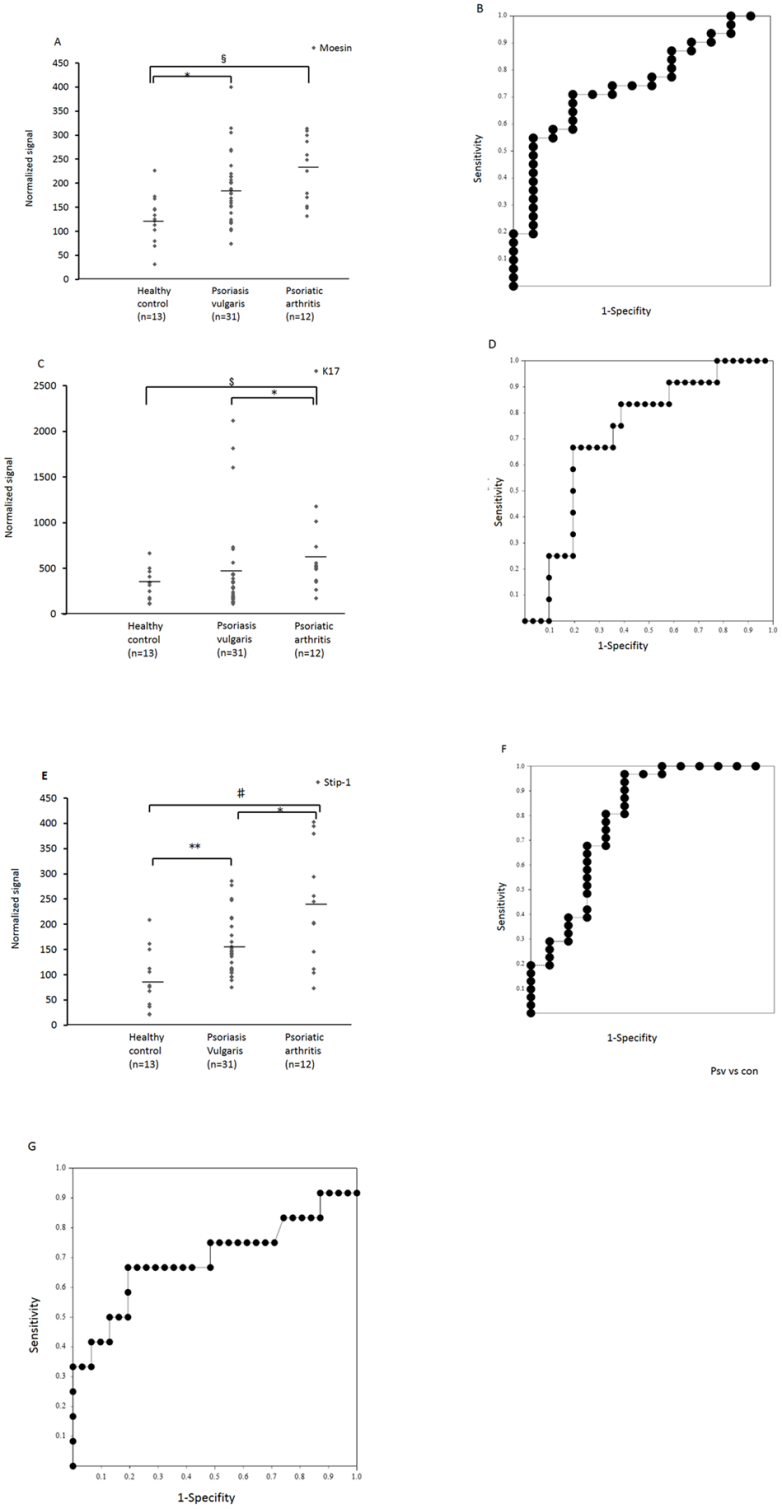
Serum moesin (A and B), K17 (C and D), and STIP1 (E, F and G) levels in patients with psoriasis vulgaris, psoriatic arthritis, and healthy controls. (A) Serum moesin levels were significantly higher in psoriasis vulgaris and psoriatic arthritis patients compared to healthy controls (*: *P*<0.05 and §: *P*<0.001, respectively). (B) Receiver-operating characteristic curve (ROC) analysis of moesin as a serum marker for psoriasis vulgaris or psoriatic arthritis. The corresponding area under the curve (AUC) was 0.747 for psoriasis vulgaris. With a 76.9% specificity, the sensitivity of moesin for psoriasis vulgaris was 71.0% at a cut-off value corresponding to 146.6. (C) Serum K17 levels were significantly higher in psoriatic arthritis than in healthy controls and psoriasis vulgaris (*: *P*<0.05, $: *P*<0.01). (D) ROC analysis of K17 as a serum marker for psoriatic arthritis. AUC was 0.72 for psoriatic arthritis. With a 80.6% specificity, the sensitivity of K17 for psoriatic arthritis was 66.7% at a cut-off value corresponding to 439.5. (E) Serum STIP1 levels were significantly higher in psoriasis vulgaris and psoriatic arthritis compared to healthy controls (*P*<0.005 each). Serum STIP1 levels were also significantly higher in psoriatic arthritis compared to healthy controls or psoriasis vulgaris (*: *P*<0.05, **: *P*<0.005, #: *P*<0.005). (F) ROC analysis of STIP1 as a serum marker for psoriatic vulgaris. AUC was 0.79 for psoriatic vulgaris. With a 69.2% pecificity, the sensitivity of Stip-1 for psoriatic arthritis was 80.7% at a cut-off value corresponding to 105.8. (G) ROC of STIP1 as a serum marker for psoriatic arthritis. AUC was 0.70 for psoriatic arthritis. With a 42.9% specificity, the sensitivity of STIP1 for psoriatic arthritis was 66.7% at a cut-off value corresponding to 195.4.

The serum levels of moesin were detected by dot blot analysis in sera from psoriasis patients and healthy controls. These levels were significantly higher in psoriasis vulgaris or psoriatic arthritis patients than in healthy controls in the training set (*P* = 0.0105, *P* = 0.0008, respectively). Relative values of serum moesin levels ranged from 73.8 to 399.8 (median: 185.6), 131.2 to 313.4 (median: 226.9), and 31.8 to 226.2 (median: 125.80) in patients with psoriasis vulgaris, psoriatic arthritis, and healthy controls, respectively (Figure 3A). The area under the curve (AUC) of the receiver operating characteristic curve (ROC) between psoriasis vulgaris and healthy controls was 0.747 (Figure 3B). When an optimal cut-off value of 146.6 for moesin was applied, the diagnostic sensitivity and specificity for psoriasis vulgaris were 71.0 and 76.9, respectively.

Serum K17 levels were significantly higher in psoriatic arthritis patients than in healthy controls (*P* = 0.009). Relative values of serum K17 levels ranged from 113.9 to 2118.9 (median: 453.8), 174.3 to 1180.9 (median: 558.8), and 111.4 to 665.6 (median: 306.2) in patients with psoriasis vulgaris, psoriatic arthritis, and healthy controls, respectively. The AUC between psoriasis patients and healthy controls was 0.81, when an optimal cutoff value of 347.8 for K17 was applied, the diagnostic sensitivity and specificity for psoriasis patients were 83.3 and 69.2, respectively. Serum K17 levels of patients with psoriatic arthritis were significantly higher than in patients with psoriasis vulgaris (Figure 3C, *P* = 0.0264). The AUC between them was 0.72 (Figure 3D). When an optimal cut-off value of 439.5 for K17 was applied, the diagnostic sensitivity and specificity for psoriatic arthritis were 66.7 and 80.6, respectively.

Serum STIP1 levels were significantly higher in patients with psoriasis vulgaris or psoriatic arthritis than in healthy controls (*P* = 0.0025, *P* = 0.0019, respectively). Relative values of serum STIP1 levels ranged from 75.2 to 286 (median: 152.5), 73.2 to 402.4 (median: 234.1), and 21.0 to 208.2 (median: 86.0) in patients with psoriasis vulgaris, psoriatic arthritis, and healthy controls, respectively (Figure 3E). The AUC between psoriasis patients and healthy controls was 0.87. When an optimal cut-off value of STIP1 was 111.8 applied, the diagnostic sensitivity and specificity for psoriatic patients were 75.0 and 76.9, respectively. Also, the AUC between psoriasis vulgaris and healthy controls was 0.79. When an optimal cut-off value of 105.8 for STIP1 was applied, the diagnostic sensitivity and specificity for psoriasis vulgaris were 80.6 and 69.2, respectively (Figure 3F). Serum STIP1 levels of patients with psoriatic arthritis were significantly higher than in patients with psoriasis vulgaris (*P* = 0.050). The AUC between patients with psoriatic arthritis and psoriasis vulgaris was 0.69. When an optimal cut-off value of 195.4 for STIP1 was applied, the diagnostic sensitivity and specificity for psoriatic arthritis were 66.7 and 42.9 (Figure 3G). However, the serum STIP1 or moesin, CK17 level was not correlated with PASI score or BSA in patients with psoriasis vulgaris or psoriatic arthritis and DAS28 or BASDAI swollen or tender joint counts, and involved joint counts in patients with psoriatic arthritis.

Relative values of serum ANXA1 levels ranged from 165.5 to 4041.0 (median: 772.6), 287.0 to 1591.0 (median: 821.3), and 226.0 to 1704.0 (median: 706.4) in patients with psoriasis vulgaris, psoriatic arthritis, and in healthy controls, respectively. However, no significant differences for serum ANXA1 levels among these groups were observed. The serum ANAX1 level was weakly correlated with the PASI score or BSA in patients with psoriasis vulgaris, but not in psoriatic arthritis. Also the serum STIP1 or moesin, CK17, ANXA1 level was not correlated with DAS28 or BASDAI in patients with psoriatic arthritis.

## Discussion

In this study, a total of 22 psoriasis-associated autoantigens, including moesin, K17, STIP1, and ANXA1, were identified employing 2D-IB with sera from patients with psoriasis.

Moesin is a protein that in humans is encoded by the *MSN* gene [11]. Moesin (membrane-organizing extension spike protein) is a member of the ERM family, which includes ezrin and radixin [12]. A previous study reported that solute carrier family 9, isoform 3 regulatory factor 1 (SLC9A3R1) is associated with susceptibility to psoriasis [13]. SLC9A3R includes a PDZ domain, which is a common structural domain of 80–90 amino-acids found in the signaling proteins of bacteria, yeast, plants, viruses, and animals. Postsynaptic density 95 (PSD-95), Drosophila discs-large tumor suppressor (Dlg), and Zonula occludens-1 (ZO-1) also include PDZ domains that associate with members of the ezrin-radixin-moesin family and are implicated in diverse aspects of epithelial membrane biology and immune synapse formation in T cells [13], [14], [15]. Thus, moesin may have a crucial role in the pathogenesis of psoriasis.

K17 is a protein that is encoded by the *KRT17* gene in humans. K17 is a type I keratin and is found in nail beds, hair follicles, sebaceous glands, and other epidermal appendages [16]. In this study K17 was found to be strongly expressed in psoriatic lesions but not in normal skin. De Jong *et al* reported that K17 expression is a hallmark of psoriasis [17]. It has been shown that IFN-γ can upregulate K17 expression by activating STAT 1 [18]. K17, which is a major target for autoreactive T cells, may function as an autoantigen in the immunopathogenesis of psoriasis [19]. Th17- and IL-22-producing CD4^+^ T cells upregulate the expression of K17 in keratinocytes. In addition, K17 stimulates autoreactive T cells and promotes the production of psoriasis-associated cytokines [20]. K17 is thus an attractive target for novel therapies aimed at curtailing psoriasis driven by chronic inflammation [16]. However, no study concerning the relationship between K17 and psoriatic arthritis or rheumatoid arthritis has been reported. To our knowledge, this is a first report showing that K17 is a possible sero-diagnostic marker for psoriatic arthritis.

STIP1, which is also called heat shock protein 70 kDa/heat shock protein 90 kDa (HSP70/HSP90)-organizing protein, is an adaptor protein that coordinates the functions of HSP70 and HSP90 in protein folding. It is thought to assist in the transfer of proteins from HSP70 to HSP90 by binding both HSP90 and substrate-bound HSP70. STIP1 also stimulates the ATPase activity of HSP70 and inhibits the ATPase activity of HSP90, suggesting that it regulates both the conformations and ATPase cycles of these chaperones [21]. STIP1 has recently been shown to be secreted by human ovarian cancer cells and is well-known as a biomarker of ovarian carcinoma [22]. In neuronal tissues, STIP1 binding is needed for the activation of the extracellular-regulated mitogen activated protein kinase (ERK) signaling pathway [23]. It is uncertain if there is a direct relationship between STIP1 and the pathogenesis of psoriasis vulgaris or psoriatic arthritis. However, STIP1 activates ERK and c-Jun N-terminal kinase (JNK), and JNK is expressed in psoriatic epidermis [24]. STIP1 might be related to the hyperproliferation and abnormal differentiation of psoriatic epidermis. Another report has shown that etanercept therapy results in a significant decrease in the levels of NFκB, JNK, and ERK in the synovia of patients with psoriasis arthritis [25]. Persistent activation of these pathways, albeit reduced, may trigger positive feedback loops and flares of psoriatic arthritis [25]. STIP1 may therefore have an important role in the pathogenesis of psoriatic arthritis through the activation of ERK and JNK. An association between HSP70 or HSP90 and psoriasis has been reported [26], [27]. HSP70, a ligand for CD91, is increased in keratinocytes in close vicinity to CD91-bearing antigen-presenting cells in psoriatic lesions [26]. Act1 is a client protein of HSP90 and IL-17 receptor adaptor protein, and is required in the IL-17 signaling cascade as a key signaling molecule; a variant of Act1 is linked to psoriasis susceptibility and has impaired interaction with HSP90 [27]. The relationship of serum IgG to HSP70 or HSP90 and psoriatic arthritis is not clear, but in patients with rheumatoid arthritis with articular erosions, serum anti-HSP 90 IgG is most common, suggesting that it might be related to articular prognosis [28]. There are no reports concerning the role of STIP1 in the pathogenesis of psoriasis; however, the present study shows it is possible that STIP1 is a sero-diagnostic marker in patients with psoriasis vulgaris or psoriatic arthritis.

ANXA1, also called as lipocortin-1, belongs to a family of Ca (2+)-dependent phospholipid-binding proteins that are preferentially located on the cytosolic face of the plasma membrane. ANXA1 has phospholipase A2 inhibitory activity. Phospholipase A2 is required for the biosynthesis of potent mediators of inflammation. ANXA1 may thus have potential anti-inflammatory activity [29]. ANXA1 is also considered to be a putative substrate of epidermal growth factor receptor/kinase, protein kinase C, and tissue transglutaminase [30]. We confirmed the expression of ANXA1 in the upper layer of psoriatic epidermis; however, a previous study reported that ANXA1 has a role in psoriatic lesion formation without necessary disease specificity [30], [31]. We tested the same result previously reported that serum ANXA1 levels in patients with psoriasis vulgaris are elevated compared to healthy controls, but no significant difference was observed [32]. Furthermore, we found for the first time that serum ANXA1 levels were weakly correlated with patients’ PASI scores and BSA. There are a few reports concerning ANXA1 and psoriatic arthritis, but no detailed mechanism of ANXA1 in psoriatic arthritis has been elucidated, although a role for ANXA1 in the synovia of rheumatoid arthritis patients has been reported [33], [34]. The role of ANXA1 in the pathogenesis of psoriatic arthritis is unclear, and further study is necessary. In this study, we analyzed serum levels of moesin, K17, STIP1, and ANXA1 in psoriasis patients and healthy controls, and measured the stainability of these proteins in psoriasis tissues. Our results suggest that moesin and STIP1 may be useful sero-diagnostic markers for psoriasis vulgaris and psoriatic arthritis.
